# New Appliances and Things Medical

**Published:** 1905-09-09

**Authors:** 


### NEW APPLIANCES AND THINGS MEDICAL.

### SERRAVALLO'S TONIC WINE.

46 HOLBORN VIADUCT, E.C.

THE preparation before us is a tonic wine containing the active principles of cinchona bark and iron along with alcohol. The dose given on the bottle is two to three liqueur glasses a day, or approximately one fluid ounce three times a day. The price of a bottle containing 18 ounces is 3s. 6d. Pharmacologically Serravallo's Wine acts as a bitter, carminative, and tonic; it is best taken about 20 minutes before meals, when it will promote the secretion of gastric juice, both reflexly from the palate and directly by stimulating the gastric mucous membrane. In addition, the iron it contains exerts a hæmatinic action. The taste is quite pleasant, the usual astringent taste of the iron being barely perceptible. We think this tonic wine is to be recommended, and should be useful in stimulating appetite, digestion, and nutrition when these functions have been exhausted by disease.

